# Single-Strain Probiotic Lactobacilli for the Treatment of Atopic Dermatitis in Children: A Systematic Review and Meta-Analysis

**DOI:** 10.3390/pharmaceutics15041256

**Published:** 2023-04-17

**Authors:** Sabina Fijan, Nina Kolč, Metka Hrašovec, Gro Jamtvedt, Maja Šikić Pogačar, Dušanka Mičetić Turk, Uroš Maver

**Affiliations:** 1 Faculty of Health Sciences, University of Maribor, Žitna ulica 15, 2000 Maribor, Slovenia; 2 Department of Pediatrics, Faculty of Medicine, University of Maribor, Taborska ulica 8, 2000 Maribor, Slovenia; 3 Faculty of Health Sciences, Oslo Metropolitan University, 0130 Oslo, Norway; 4 Institute of Biomedical Sciences, Faculty of Medicine, University of Maribor, Taborska ulica 8, 2000 Maribor, Slovenia; 5 Department of Pharmacology, Faculty of Medicine, University of Maribor, Taborska ulica 8, 2000 Maribor, Slovenia

**Keywords:** probiotics, lactobacilli, atopic dermatitis, children, meta-analysis, systematic review

## Abstract

Probiotics are known for their positive effects on the gut microbiota. There is growing evidence that the infant gut and skin colonization have a role in the development of the immune system, which may be helpful in the prevention and treatment of atopic dermatitis. This systematic review focused on evaluating the effect of single-strain probiotic lactobacilli consumption on treating children’s atopic dermatitis. Seventeen randomized placebo-controlled trials with the primary outcome of the Scoring Atopic Dermatitis (SCORAD) index were included in the systematic review. Clinical trials using single-strain lactobacilli were included. The search was conducted until October 2022 using PubMed, ScienceDirect, Web of Science, Cochrane library and manual searches. The Joanna Briggs Institute appraisal tool was used to assess the quality of the included studies. Meta-analyses and sub meta-analyses were performed using Cochrane Collaboration methodology. Due to different methods of reporting the SCORAD index, only 14 clinical trials with 1124 children were included in the meta-analysis (574 in the single-strain probiotic lactobacilli group and 550 in the placebo group) and showed that single-strain probiotic lactobacilli statistically significantly reduced the SCORAD index compared to the placebo in children with atopic dermatitis (mean difference [MD]: −4.50; 95% confidence interval [CI]: −7.50 to −1.49; Z = 2.93; *p* = 0.003; heterogeneity I^2^ = 90%). The subgroup meta-analysis showed that strains of *Limosilactobacillus fermentum* were significantly more effective than strains of *Lactiplantibacillus plantarum*, *Lacticaseibacillus paracasei* or *Lacticaseibacillus rhamnosus*. A longer treatment time and younger treatment age statistically significantly reduced symptoms of atopic dermatitis. The result of this systematic review and meta-analysis shows that certain single-strain probiotic lactobacilli are more successful than others in reducing atopic dermatitis severity in children. Therefore, careful consideration to strain selection, treatment time and the age of the treated patients are important factors in enhancing the effectiveness of reducing atopic dermatitis in children when choosing probiotic single-strain lactobacilli.

## 1. Introduction

Atopic dermatitis (AD) is a chronic inflammatory skin condition. It is the most common type of eczema that can occur at any age but is the most common in children. It is a heterogeneous disorder with various associated manifestations and symptoms. Cases may range from mild to severe. Worldwide, approximately 2 million children suffer from AD, which has a lifetime prevalence as high as 20% and that continues to rise [1,2,3,4].

The best and most commonly used validating scoring system in AD is the SCORAD index (SCORing Atopic Dermatitis) [5], which was developed in 1993 by the European Taskforce on Atopic Dermatitis (ETFAD). It is based on the formula A/5 + 7B/2 + C, where A is defined as the extent (0–100), B is defined as the intensity (0–18) and C is defined as the subjective symptoms (0–20). The maximal score of the SCORAD Index is 103. The extent is graded from 0–100 and applied on a front/back drawing of the patient’s inflammation lesions. The intensity part of AD consists of six major features: erythema, oedema/papulation, oozing crusts, excoriations, lichenification and dryness. Each item can be graded on a scale of 1–3. The subjective symptoms (maximum score 20) include daily pruritus and sleeplessness. Other measurement scales include the Eczema Area and Severity Index (EASI), the objective component of SCORAD (oSCORAD), the modified EASI (mEASI), the Atopic Dermatitis Severity Index (ADSI), body surface area (BSA), and three-item severity score (TIS), among others. AD is graded as mild, moderate or severe based on the SCORAD index of under 25, between 25 and 50 and above 50, respectively [6,7].

The pathogenesis of AD is not well understood. However, the role of the skin microbiome and the intestinal microbiome in promoting normal immune system functions and preventing the colonization of pathogens is being elucidated [1,8]. The apparent increase in atopic disease, particularly in food allergy, over the past 2 decades has resulted in reconsidering prevention strategies aimed at the infant’s diet. Early advice that suggested to have atopy-prone infants delay the ingestion of potential food allergens, such as eggs, cow’s milk, and peanuts, was rescinded, as new evidence emerged that did not support these approaches. More recently, randomized controlled trials have provided data to support an opposite strategy, promoting the early ingestion of allergens as a means of food allergy prevention [9]. Over the last 10 years, nearly half of all clinical studies investigated the efficacy and safety of novel therapeutic agents, particularly biologics and small molecules. Other clinical studies included skin moisturizers and probiotics. The latter focus on the skin and gut microbiome’s role in preventing or treating AD [10]. Additional dietary approaches regarding breastfeeding, the early introduction of other types of food allergens, formula feeding, dietary nutrients and probiotics are also under scrutiny as potential preventative strategies [9].

Probiotics are ‘live microorganisms that, when administered in adequate amounts, confer a beneficial effect on the host’ [11]. The most common probiotics are members of the lactobacilli group, which has recently been divided into 23 novel genera [12]. The most common lactobacilli that contain probiotic strains are strains of the following species: *Lacticaseibacillus rhamnosus*, *Lacticaseibacillus casei*, *Limosilactobacillus reuteri*, *Limosilactobacillus fermentum*, *Lactiplantibacillus plantarum*, *Latilactobacillus sakei*, *Levilactobacillus brevis*, *Lactobacillus acidophilus*, *Lactobacillus gasseri*, *Lactobacillus delbrueckii* subsp. *bulgaricus* and many more. The next most used probiotics are from the genus *Bifidobacterium* genera (e.g., *Bifidobacterium infantis*, *Bifidobacterium animalis* subsp. *lactis*, and *Bifidobacterium longum*). In addition, strains from other bacterial species (e.g., *Pediococcus acidilactici*, *Lactococcus lactis*, *Leuconostoc mesenteroides*, *Enterococcus faecium*, *Streptococcus thermophilus*, *Bacillus subtilis*, *Bacillus coagulans*, *Clostridium butyricum*, *Propionibacterium freudenreichii* and *Escherichia coli*) and certain yeasts (e.g., *Saccharomyces cerevisiae* var. *boulardii*) qualify as probiotics [13]. Probiotics have been shown to be efficient therapeutics for various diseases and conditions, including skin conditions, inflammatory bowel disease and other gastrointestinal conditions, as noted by recent systematic reviews and meta-analyses [14,15,16,17]. The current use of probiotics relies on several proven therapeutic properties or mechanisms. These include their antimicrobial activity, competitive exclusion, immunomodulation, improvement of intestinal barrier function, production of beneficial metabolites, and improvement of cognitive function, as well as their anti-diabetic, anti-obesity, anti-cancer, and anti-allergic activities, and many more [15,18,19,20,21,22,23]. The efficiency of probiotics is strain specific, which means that a clinical study to establish a health benefit evaluation must be made for every single one of them, and, in most cases, benefits cannot be generalized. On the other hand, different probiotic strains have different levels of effectiveness for a particular health ailment, and it is not possible to generalize the health benefits of whole classes of probiotics [11,24].

Probiotics are a promising means of beating the allergy epidemic with the underlying concept being based on the modulation of the gut microbiota and the development of infant immunity [25]. Several recent reviews and meta-analyses on probiotics’ efficacy in treating or preventing AD have been published to date [26,27,28,29,30,31,32]. The review by Anania and co-authors [26] emphasizes the proven immunomodulatory effects of probiotics and the production of short-chain fatty acids that aid in preserving immune homeostasis as well as the modulation of the maternal gut microbiota in infant microbiota via the administration of probiotics during pregnancy and lactation. Similarly, Liu and co-authors [27] concluded that gut microbiota changes are essential to the development of AD in children and may be an effective target for the prevention and treatment of AD. Boggio and co-authors [28] focused on *Lacticaseibacillus rhamnosus* GG (previously known as *Lactobacillus rhamnosus* GG) and its role in pediatrics. They found that the early administration of this strain during pregnancy reduced the development of AD in the infant. Jiang and co-authors [29] concluded that intervention with probiotics potentially lowered the incidence of AD and relieved symptoms of AD in children, particularly when treating infants and children over one year of age. Their sub-group analysis showed that both single-strain and mixed-strain probiotics significantly affected SCORAD values. Similarly, Sun and co-authors [30] found that probiotics seemed effective against atopic eczema after 1 year of age. On the other hand, D’Elios and co-authors [31] concluded that the effects of probiotic administration for the prevention/treatment of allergic diseases and AD are still so controversial that no definitive recommendation can be made at this stage. Similarly, Huang and co-authors [32] concluded that research has not robustly shown that probiotics benefit children with AD. These conclusions are perhaps due to assessing all the vast different probiotic species and strains as one group. Most authors of these reviews also concluded that, although the results of clinical studies are promising, the comparison is limited due to the heterogeneity among the studies, which include diversity in the type, dose and timing of probiotics administration as well as in the period of follow-ups after treatment [26,29,31,32].

None of these reviews or meta-analyses has focused only on the effect of single-strain lactobacilli. The efficiency of probiotics can be strain specific as is established from the published reviews; however, this angle has not been separately addressed in the above-mentioned reviews. Therefore, the following systematic review and meta-analysis aim to determine whether the supplementation with single-strain probiotic lactobacilli for treating AD in children decreases the SCORAD index compared to a placebo.

We formulated the research according to the PICO strategy (Population, Intervention, Comparison, Outcome) compilation [33], and asked, are single-strain probiotic lactobacilli (I), compared to placebos (C), given to the child (P) effective in reducing atopic dermatitis (O)?

## 2. Materials and Methods

### 2.1. Search Strategy and Selection Criteria

The present review’s design, structure and reporting conform with the Preferred Reporting Items for Systematic Reviews and Meta-Analyses (PRISMA) guidelines [34]. The PRISMA checklist is presented in Supplementary S1. The electronic databases PubMed/MEDLINE, ScienceDirect, Cochrane Library and Web of Science were searched by three independent reviewers (SF, MK and NK) from July 2022 to October 2022 using the following key words: “probiotics” or “*Lactobacillus*” and “atopic dermatitis” or “eczema”. We searched for the following Medical Subject Headings (MeSH): (Probiotic OR *Lactobacillus*) AND (Atopic Dermatitis OR Eczema). The search was restricted to children using the limits “Humans” and “Child: birth–18 years”. Additional studies that were included in the reference list were searched manually. All the studies retrieved from different databases were imported to Endnote 20 (Clarivate Analytics, Chandler, AZ, USA), and all duplicates were removed. Any differences were resolved by discussion among the authors of this article.

### 2.2. Eligibility Criteria

We included all published double-blind, randomized and placebo-controlled trials (RCTs) involving children and adolescents (aged 0–18 years) that evaluated the effect of probiotic single-strain lactobacilli on treating AD. Participants were prohibited from consuming any prebiotic, synbiotic, heat-killed probiotic or systemic corticosteroid. Trials needed to define their patient enrolment, the daily dosage of probiotics intake, the type of ingested probiotics, the placebo, the route of administration (only studies with oral administration were included) and the results of the SCORAD index, either reported before and after treatment or as mean difference.

### 2.3. Data Extraction and Critical Assessment

Three authors (NK, MK and SF) independently screened articles by considering the abstracts and full text. Data extraction included the first author, the year of publication, the number and age of participants, the type and dosage of probiotics used, study duration, study outcome and the SCORAD index of each study.

The methodology of the studies was assessed using the Joanna Briggs Institute critical appraisal tool (JBI) for randomized controlled trials [35]. Based on Camp and Legge’s [36] recommendation, we evaluated the studies as medium-high quality (70–79%), high quality (80–90%) and excellent quality (90% or more). The authors MŠP and SF conducted this critical assessment.

### 2.4. Statistical Analysis

The author SF entered data into Review Manager software (RevMan) and performed statistical analyses using Cochrane’s Review Manager [37]. We analyzed continuous data using mean differences (MDs) and reported the 95% CI on all estimates using the random effects model based on the DerSimonian method. If the SCORAD index data were not reported in mean and standard deviation, we used standard statistical methods to convert the data as follows: for calculating the difference as mean difference and standard deviation (SD) of the SCORAD index before and after treatment (SD was calculated from the 95% confidence interval), we used the Cochrane Handbook for Systematic Reviews of Interventions [38]; for calculating the mean and SD from results reported as the median and interquartile range, we used the models to estimate the sample mean and standard deviation [39,40]. We used the random-effects model for all meta-analyses and assessed the heterogeneity between the included trials using the I^2^ statistic. The degree of heterogeneity was graded as non-existent or minimal for an I^2^ value of less than 25%, low for an I^2^ value of 25–49%, moderate for an I^2^ value of 50–74%, and high for an I^2^ value of 75–100%.

## 3. Results

### 3.1. Study Selection

**Figure 1:**
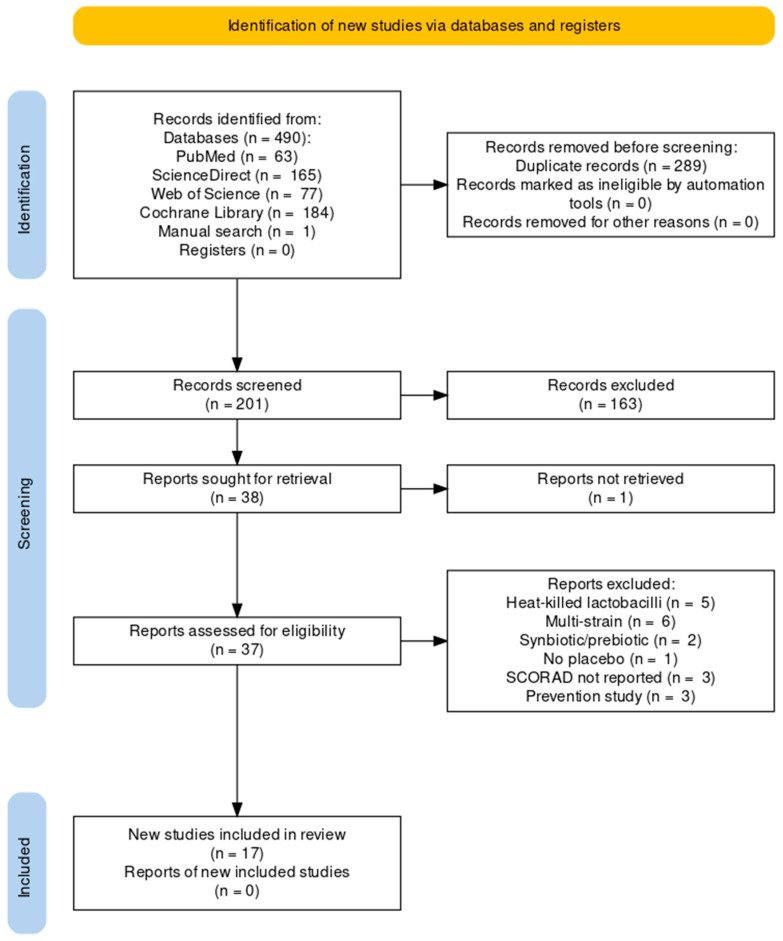
PRISMA flow chart of the study selection process.

A total of 490 articles were identified based on the described methodology, and 201 articles remained after removing duplicates. After the screening of titles and abstracts, 163 records were excluded. One study was unavailable as full text, and thirty-seven were assessed for eligibility. After excluding 20 studies (heat-killed lactobacilli, multi-strain probiotics, synbiotics or prebiotics were used; no SCORAD was reported; no placebo was used; or the study investigated preventative effects only), 17 studies were included and abbreviated as the first author and the publication year as follows: Isolauri 2000 [41], Kirjavainen 2003 [42], Viljanen 2005 [43], Weston 2005 [44], Brouwer 2006 [45], Fölster-Holst 2006 [46], Grüber 2007 [47], Woo 2010 [48], Klewicka 2011 [49], Gore 2012 [50], Han 2012 [51], Wang 2015 [52], Prakoeswa 2017 [53], Wu 2017 [54], Ahn 2020 [55], Rather 2021 [56] and Carucci 2022 [57]. The flow chart of the study selection is presented in Figure 1. The Joanna Briggs Institute critical appraisal tool for randomized controlled trials [35] was used to assess the quality of the clinical trials. The assessment is noted in Table 1.

The quality of the trials was assessed using the following questions: 1. Was true randomization used for assignment of participants to treatment groups? 2. Was allocation to treatment groups concealed? 3. Were treatment groups similar at the baseline? 4. Were participants blind to treatment assignment? 5. Were those delivering treatment blind to treatment assignment? 6. Were outcomes assessors blind to treatment assignment? 7. Were treatment groups treated identically other than the intervention of interest? 8. Was follow up complete and, if not, were differences between groups in terms of their follow-up adequately described and analyzed? 9. Were participants analyzed in the groups to which they were randomized? 10. Were outcomes measured in the same way for treatment groups? 11. Were outcomes measured in a reliable way? 12. Was appropriate statistical analysis used? 13. Was the trial design appropriate and were any deviations from the standard RCT design (individual randomization, parallel groups) accounted for in the conduct and analysis of the trial? The possible answers were YES, NO, unclear and not applicable (N/A). The study quality was rated according to Camp and Legge’s recommendation [36].

Eight studies [43,44,47,50,51,52,56,57] were rated excellent quality as at least twelve of thirteen questions were assessed as positive. Four studies [46,49,54,55] were rated high quality with two unclear answers, while the remaining five were rated as medium-high. In four [41,45,48,53] of the latter five studies, three questions were rated unclear, and in one study [42], one question was rated as ‘no’ as some of the infants included in the study were assigned to open-label with regard to the cow’s milk challenge. However, we included the study as the SCORAD score was measured. Questions Q1 and Q2 regarding the randomization and concealment of allocation were rated as unclear in several studies as the authors did not specifically report this data. Treatment groups were similar at baseline (Q3) when focusing on SCORAD scores for all studies. Seven studies [46,47,50,51,52,54,57] specifically stated that intention-to-treat analysis was used as noted in question Q9 and, therefore, received a positive score for this question.

### 3.2. Characteristics of the Included Studies

The 17 included clinical trials [41,42,43,44,45,46,47,48,49,50,51,52,53,54,55,56,57] were published between 2000 and 2022. Eight studies were conducted in Europe [41,42,43,45,46,47,49,57], seven studies in Asia [48,52,53,54,55,56], while one study was conducted in New Zealand [50] and one was conducted in Australia [44]. All 17 studies were placebo-controlled and double-blind clinical trials. The outcome in all 17 studies was reported using the SCORAD index and either reported before and after treatment [41,42,46,47,48,49,50,52,53,55], as a mean difference [43,44,54,56], reported either before or after treatment and as a mean difference [51], or graphically [45,57].

Table 2 summarizes the overall characteristics of the included studies. These are as follows: the population that completed the trial, divided into the probiotic group and the placebo group; the intervention parameters of single-strain probiotic lactobacilli (including the probiotic strain, the concentration measured in cfu and the daily supplementation); the duration of supplementation; the main findings regarding the SCORAD index; and the findings on immunological parameters and intestinal microbiota.

Most of the studies investigated the influence of *Lacticaseibacillus rhamnosus* GG for treating AD [41,42,43,45,46,47,57]. Only two found a beneficial effect [41,42], while five did not [43,45,46,47,57]. One study [54] investigated another strain, *Lacticaseibacillus rhamnosus* MP108, and found a statistically significant lower SCORAD index. Two *Latilactobacillus sakei* strains, KCTC 10755BP [48] and proBio65 [56], were investigated, and both found a statistically significant lower SCORAD index of patients with AD for the probiotic group compared to placebo. In addition, both investigated *Limosilactobacillus fermentum* strains VRI-033 PCC [44] and GM090 [52], and both investigated *Lactiplantibacillus plantarum* strains CJLP133 [51] and IS-10506 [53], which were also successful in statistically significantly lowering the SCORAD index. Two *Lacticaseibacillus paracasei* strains were investigated: CNCM [50] and GMNL-133 [52]. The latter exhibited a statistically significant lower SCORAD index, while the former did not. The investigated probiotic *Lacticaseibacillus casei* DN-114001 [49] achieved a statistically significant reduction in SCORAD index in the probiotic group compared to the placebo. Most studies involved a supplementation with single-strain probiotic lactobacilli for 3 months or 12 weeks [45,47,48,49,50,51,52,53,55,56,57]. Four studies involved supplementation for 7.5 or 8 weeks or 2 months [41,42,44,46,54], and one study involved shorter supplementation, namely, 4 weeks [43].

### 3.3. Meta-Analysis of the Effect of Single-Strain Probiotic Lactobacilli for the Treatment of Atopic Dermatitis

**Figure 2:**
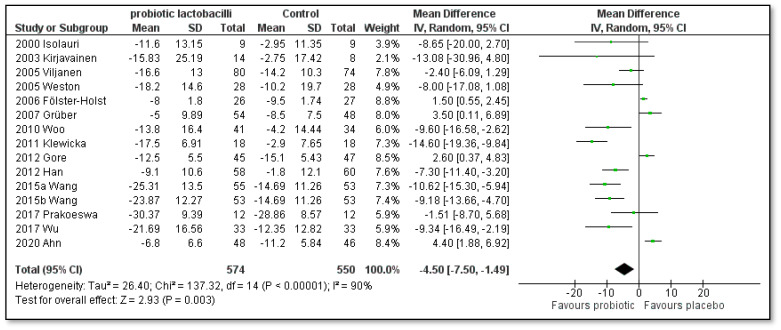
Meta-analysis of the effect of single-strain probiotic lactobacilli compared to placebo on the SCORAD index change in children with AD [41,42,43,44,46,47,48,49,50,51,52,53,54,55].

Brouwer 2006 [45], Rather 2021 [56] and Carucci 2022 [57] could not be included in the meta-analysis as the SCORAD index was depicted differently, namely, in graphical form in fixed predicted values, in graphical form as means and error bars, and in a graphical form showing the percentage of children with a reduction in more than 8.7 units. A total of 11 trials [41,42,46,47,48,49,50,51,52,53,55] reported the SCORAD index at baseline and after treatment, and five trials [43,44,51,54,56] reported a decrease in the SCORAD index after treatment. Of these, the trial by Han and co-authors [51] also reported the above-mentioned SCORAD index at baseline and after treatment. All 14 trials have depicted SCORAD index changes in the forest plot, as shown in Figure 2. The trial by Wang 2015 [52] separately investigated two single-strain probiotics, which are shown separately. The meta-analysis for the outcome of the difference in the SCORAD index of children with AD is also noted in Figure 2.

**Figure 3:**
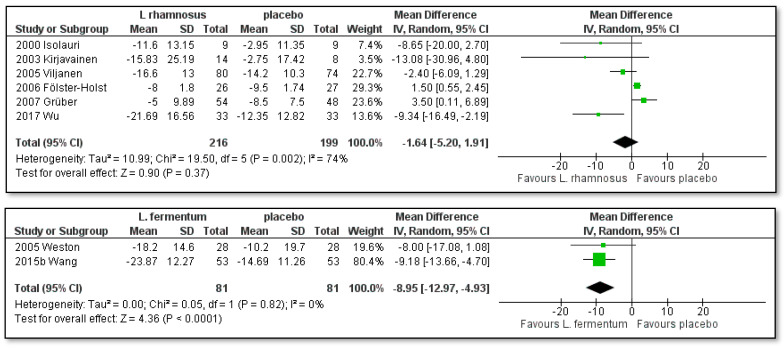

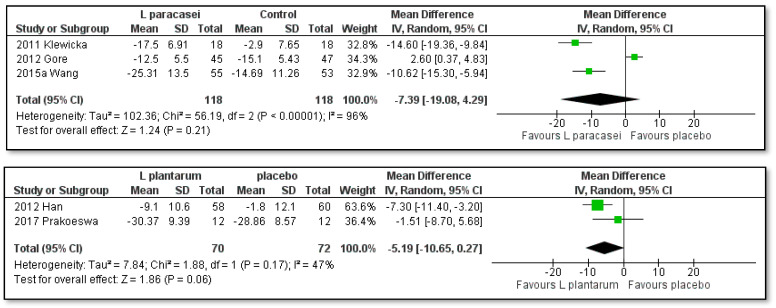
Subgroup meta-analyses of the effect of the included single-strain probiotic *Lacticaseibacillus rhamnosus*, *Limosilactobacillus fermentum*, *Lacticaseibacillus paracasei* and *Lactiplantibacillus plantarum* strains compared to placebo on the SCORAD index change in children with AD [41,42,43,44,46,47,49,50,51,52,53,54].

**Figure 4:**
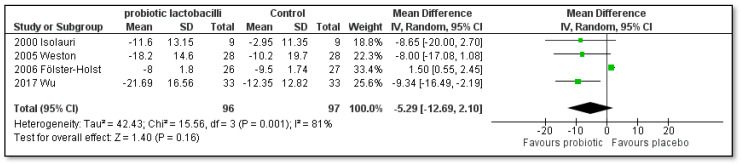
Subgroup meta-analyses of the effect of 8 weeks treatment time with single-strain probiotic lactobacilli compared to placebo on the SCORAD index change in children with AD [41,44,46,54].

**Figure 5:**
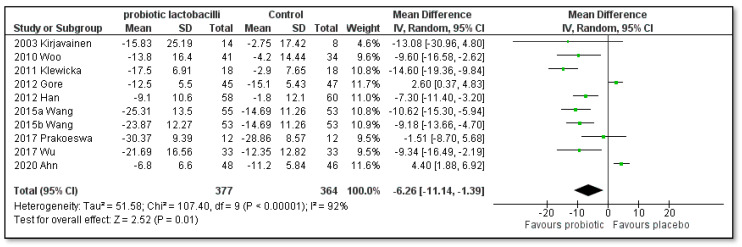
Subgroup meta-analyses of the effect of 12 weeks treatment time with single-strain probiotic lactobacilli compared to placebo on the SCORAD index change in children with AD [42,48,49,50,51,52,53,54,55].

**Figure 6:**
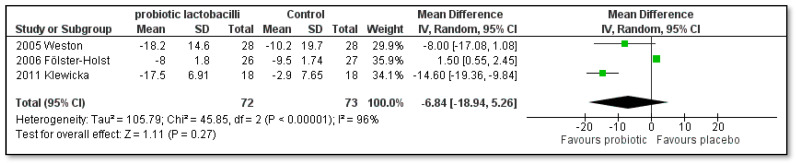
Subgroup meta-analyses of the effect of single-strain probiotic lactobacilli compared to placebo on the SCORAD index change in children with moderate to severe AD [44,46,49].

**Figure 7:**
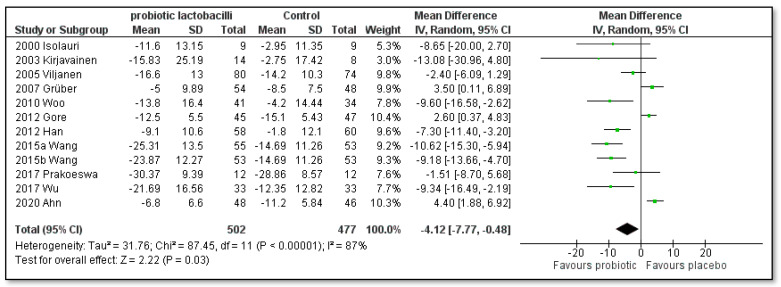
Subgroup meta-analyses of the effect of single-strain probiotic lactobacilli compared to placebo on the SCORAD index change without studies of children with moderate to severe AD [41,42,43,47,48,50,51,52,53,54,55].

**Figure 8:**
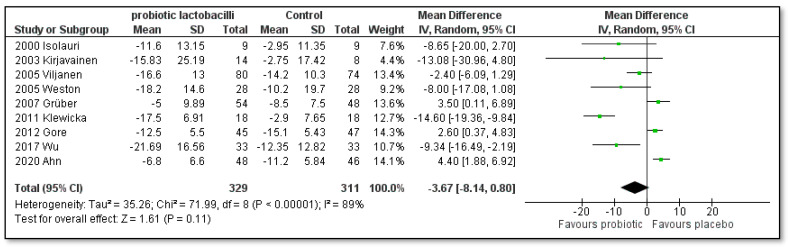
Subgroup meta-analyses of the effect of single-strain probiotic lactobacilli compared to placebo on the SCORAD index change in children up to 4 years of age [41,42,43,44,47,49,50,54,55].

**Figure 9:**
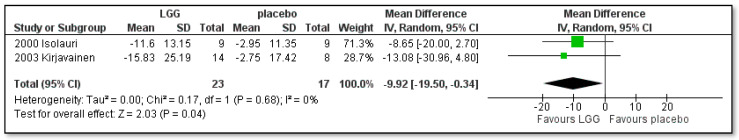
Subgroup meta-analyses of the effect of 3 months supplementation with *Lacticaseibacillus rhamnosus* GG (LGG) compared to placebo on the SCORAD index change in children with AD aged under 1 year at treatment time [41,42].

A total of 1.124 children were assessed (574 in the single-strain probiotic lactobacilli group and 550 in the placebo group). A statistically significant difference was found in favor of all included single-strain probiotic lactobacilli compared to placebo (mean difference [MD]: −4.50; 95% confidence interval [CI]: −7.50 to −1.49; Z = 2.93; *p* = 0.003; heterogeneity I^2^ = 90%). However, as high heterogeneity was found, we divided the studies into subgroups according to species (Figure 3), eight weeks of treatment time (Figure 4), twelve weeks of treatment time (Figure 5), children with or without initial moderate to severe AD (Figure 6 and Figure 7), if single-strain probiotic lactobacilli were consumed at the age of up to 4 years (Figure 8) and if LGG was consumed at the age of up to 1 year (Figure 9).

As shown in Figure 3, the sub-analysis of single-strain probiotic lactobacilli has resulted in a statistically significant effect of the SCORAD index change in children with AD after treatment with the investigated probiotic strains of *Limosilactobacillus fermentum* compared to placebo ([MD]: −8.95; [95% CI]: −12.97 to −4.93; Z = 4.36; *p* = 0.0001; I^2^ = 0%) with low heterogeneity. The studies by Weston and Wang [44,52] investigated two strains of *Limosilactobacillus fermentum*, namely, VRI-033 PCC and Lf GM090.

On the other hand, the investigated probiotic strains of *Lacticaseibacillus paracasei* ([MD]: −7.39; [95% CI]: −19.08 to 4.29; Z = 1.24; *p* = 0.21; I^2^ = 96%), *Lactiplantibacillus plantarum* ([MD]: −5.19; [95% CI]: −10.65 to 0.27; Z = 1.86; *p* = 0.06; I^2^ = 47%) and *Lactocaseibacillus rhamnosus* ([MD]: −1.64; [95% CI]: −5.20 to 1.91; Z = 0.90; *p* = 0.37; I^2^ = 74%) compared to placebo were not effective as no significant reduction in the SCORAD index was achieved. Three *Lacticaseibacillus paracasei* strains, namely, Lpc DN-114001, Lpc CNCM I-2116 and Lpc GMNL-133, were investigated by Klewicka, Gore, Wang and their co-authors [49,50,52]. Han and Prakoeswa [51,53] investigated two strains of *Lactiplantibacillus plantarum*, namely, CJLP133 and Lpl IS-10506.

The clinical trials by Isolauri, Kirjavainen, Viljanen, Fölster-Holst, Grüber, and Wu and their co-authors [41,42,43,46,47,54] investigated probiotic strains of *Lactocaseibacillus rhamnosus*. All of the studies investigated the influence of *Lacticaseibacillus rhamnosus* GG except one [54], which investigated the influence of *Lacticaseibacillus rhamnosus* MP108. The studies by Ahn and Woo [48,55] were not included in the sub-analysis as both investigated an individual strain of the species, namely, *Latilactobacillus sakei* and *Lactiplantibacillus pentosus*, respectively.

As shown in Figure 4 and Figure 5, the sub-analysis regarding the treatment time of eight weeks ([MD]: −5.29; [95% CI]: −12.69 to 2.10; Z = 1.40; *p* = 0.16; I^2^ = 81%) did not result in a statistically significant decrease in the SCORAD index in the single-strain probiotic lactobacilli groups compared to the placebo group. However, if treatment was conducted for twelve weeks ([MD]: −0.26; [95% CI]: −11.14 to −1.39; Z = 2.52; *p* = 0.01; I^2^ = 92%), a statistically significant difference in the SCORAD scores in favor of the single-strain probiotics was indeed found. The study by Viljanen and co-authors [43] was not included in this sub-analysis as the treatment time was only 4 weeks.

As shown in Figure 6 and Figure 7, the severity of AD did influence the effect of probiotics. Figure 6 shows three clinical trials by Weston, Fölster-Holst and Klewicka and their co-authors [44,46,49], in which only children with moderate to severe symptoms of AD were recruited. No significant difference was found in favor of the single-strain probiotic lactobacilli compared to the placebo ([MD]: −6.84; [95% CI]: −18.94 to 5.26; Z = 1.11; *p* = 0.27; I^2^ = 96%). On the other hand, the subgroup meta-analysis, shown in Figure 7, found that the treatment with single-strain probiotic lactobacilli compared to the placebo achieved a statistically significant decrease in the SCORAD index in children with mild to moderate symptoms of AD ([MD]: −4.12; [95% CI]: −7.77 to −0.48; Z = 2.22; *p* = 0.03; I^2^ = 87%); however, high heterogeneity was observed.

Figure 8 depicts the influence of children with AD under 4 years consuming single-strain probiotic lactobacilli. No significant difference was found ([MD]: −3.67; [95% CI]: −8.14 to 0.80; Z = 1.61; *p* = 0.11; I^2^ = 89%). On the other hand, Figure 9 shows a statistically significant difference in favor of *Lactobacillus rhamnosus* GG if used as a treatment for children with AD under 1 year of age and for 3 months ([MD]: −9.92; [95% CI]: −19.50 to −0.34; Z = 2.03; *p* = 0.04; I^2^ = 0%).

## 4. Discussion

Our systematic review and meta-analysis show that the supplementation with single-strain probiotic lactobacilli can reduce the SCORAD index in children with AD. However, high heterogeneity was observed (90%) as the populations of included children were between 0 and 18 years of age, the children were recruited from different geographical regions, different single-strain probiotic lactobacilli were investigated, the treatment time varied, and the severity of AD in children also varied from mild to severe. The results of our subgroup meta-analyses showed that the efficacy of the single-strain probiotic lactobacilli was statistically significantly influenced by various factors, including strain selection, the duration of treatment and the age of children receiving treatment. A meta-analysis by Huang et al. [32] also suggested an overall benefit of probiotics supplementation in children with AD. Their analysis showed that probiotics effectively reduced SCORAD values in children aged 1–18 years. However, they also detected high heterogeneity among the studies and concluded that more randomized controlled trials with larger samples are necessary to identify the optimal species, dose and treatment duration for children with AD. Similarly, the meta-analysis by Jiang et al. [29] concluded that probiotics potentially lower the incidence of AD and relieve AD symptoms in children and that more powerful randomized controlled trials using standardized measurements should be conducted to assess the long-term effects of probiotics. The meta-analysis by Kim et al. [58] found that probiotics could be an option for treating AD, especially for moderate to severe AD in children and adults. However, no evidence was found supporting the beneficial role of probiotics in infants.

It is well known that probiotic lactobacilli influence immune modulation. Several studies have shown that supplementation for 3 months is more effective than for 2 months in establishing strong immunological support and counteracting inflammatory responses beyond the intestinal milieu inflammation. A possible explanation might lie in enhancing the generation of interleukin-10. In atopy, IL-10 is thought to mediate anti-inflammatory effects partly via its downregulatory effect on cytokines and the IgE switch [41,59,60,61].

One of the most important factors influencing the effectiveness of the probiotics on AD in children was strain selection, as was evident in the subgroup meta-analyses. Two studies [44,52] investigated two strains of *Limosilactobacillus fermentum*, namely, VRI-033 PCC and Lf GM090, and a statistically significant difference with low heterogeneity (*p* = 0.0001; I^2^ = 0%) was found in favor of both strains. Another review also supports this finding, as *Limosilactobacillus fermentum* strains displayed curative properties against AD in children [32]. *Limosilactobacillus fermentum* strains, which are used in the food industry as food preservatives and contribute to flavor, texture and health-promoting ingredients including antimicrobial peptides, have displayed the ability to enhance immunologic response, decrease the level of bloodstream cholesterol and prevent community-acquired gastrointestinal and upper respiratory tract infections [62,63,64].

Five clinical trials [41,42,43,46,47] investigated the well-known probiotic strain *Lactocaseibacillus rhamnosus* GG (also known as ATCC 53103 and LGG). However, no statistically significant difference was found in favor of this strain; however, a high heterogeneity was observed, which might have influenced the outcome. On the other hand, when considering only studies that treated children under 1 year of age and a treatment duration of 3 months, a statistically significant difference was found in favor of LGG. It has to be noted that in this subgroup analysis only two studies were included [41,42], which gave us a limited sample size as well as potential direct comparisons.

LGG is a well-known probiotic that modifies changes related to allergic inflammation, including inducing systematically detectable low-grade inflammation and enhancing the generation of interleukin-10, which could affect clinical effects in children [41,43,59,65,66]. Although Kim et al. [58] found no evidence supporting the beneficial role of various probiotics in infants, our subgroup meta-analysis, focusing on strain-specific analysis, supports the beneficial role of LGG on AD, proving once more how important strain selection is.

Meta-analyses of clinical trials have observed the efficacy of multi-strain probiotics, which seem more effective in preventing AD symptoms. However, both single-strain and multi-strain probiotics are effective in the curative effects of AD symptoms [29,32]. A large study and the follow-up studies by Wickens and co-authors [67,68] have shown that perinatal supplementation with a bifidobacteria strain, namely, *Bifidobacterium animalis* subsp. *Lactis* HN001, was effective in establishing a preventive effect against AD in children, while the investigated lactobacilli strain, namely, *Lacticaseibacillus rhamnosus* HN001, was more effective in preventing eczema in children [69]. Many other studies investigated the preventative effect of probiotics on AD [70,71,72]. Furthermore, several studies have investigated the treatment of AD in children with regard to other single-strain probiotics [73,74,75]; multi-strain probiotics [76,77,78,79,80,81]; postbiotics, also referred to as heat-killed probiotics [56,82,83,84,85]; synbiotics; and prebiotics [86,87,88,89]. However, these were not included in our review to allow us to conduct a more homogenous analysis of the currently available clinical data on single-strain lactobacilli alone.

The subgroup analysis of the treatment groups that consumed single-strain probiotic lactobacilli for 3 months vs. the treatment groups that consumed probiotic lactobacilli for 2 months also found a statistically significant difference in AD in children that consumed the probiotics for 3 months (*p* = 0.01; I^2^ = 92%). No significant difference was found in those clinical studies in which the children consumed probiotics for 2 months (*p* = 0.16; I^2^ = 81%). Although the heterogeneity was high, indicating the variability among studies, the random effects model was used with which we tried to balance the variability. This is contrary to the results of the meta-analysis by Zhao et al. that focused on infants [90] and found that a treatment time longer than 8 weeks did not bring any additional benefits. This might be explained by the natural progression of AD, which diminishes the effect of any treatment over time. However, as shown in various studies (including ours), a 3-month probiotic treatment is the minimum required (especially when using lactobacilli) to impact the immune system. The expected effects include the reduction in pro-inflammatory responses; the modulation of the maturation of anti-inflammatory cytokines, such as Il-10; and the stimulation of the mucosal IgA levels after colonizing and balancing the gut microbiome [91,92].

Probiotics are among the possible prevention strategies for AD. Although several studies reveal a significant reduction in AD incidence with prenatal and/or postnatal probiotic supplementation, they differ in the strains, timing, dose, treatment duration and measurement of clinical outcomes. Consequently, no firm guidelines or recommendations exist for probiotic use in pregnancy or infancy to prevent AD. Currently, probiotics may be promising, but there is inadequate data to determine their overall efficacy unequivocally [93,94]. These findings may be partially attributed to the fact that all probiotics included in various meta-analyses are not comparable, as many traits are not only species specific but also strain specific [11,24], thus preventing a generalized health benefit of different strains. Similarly, when studying meta-analyses of the effect of antibiotics against a disease, they may focus on a specific antibiotic [95], while in other meta-analyses, several antibiotics are evaluated, and some are found to be more effective than others [96,97].

The World Allergy Organization–McMaster University Guidelines for Allergic Disease Prevention also contained conflicting statements regarding probiotics in 2015 [98]. On the one hand, it was found that current evidence does not indicate that probiotic supplementation reduces the risk of developing allergies in children. On the other hand, the panel suggests using probiotics in pregnant women at a high risk for having an allergic child, in women who breastfeed infants at a high risk of developing allergies and in infants at a high risk of developing allergies [99].

The overall quality of the included studies was mixed, mostly due to missing information regarding the randomization and blinding process. However, this does not mean that the authors did not use some form of computerized randomization and that the probiotic and placebo formulations were similar in appearance, taste, smell and packaging. Furthermore, several clinical studies did not specify if the intention-to-treat (ITT) analysis was utilized. The ITT analysis is a type of statistical analysis recommended in the Consolidated Standards of Reporting Trials statement on best practices in trial reporting. It is considered a marker of good methodological quality in the analysis of results of a randomized trial [35]. Therefore, it is important that authors of clinical studies report all information to ensure the lowest possible risk of bias and the highest quality of analysis. The strength of our meta-analysis is, among others, that we included only studies with a quality score of medium-high or more and, therefore, added additional weight to the conclusions.

An important limitation of our meta-analysis is the small sample sizes of studies and the variable populations (different ages of participants). Further studies with more adequately powered RCTs using standardized measurements are necessary to assess which species of probiotics, what dosages and what length of treatment are needed to strengthen the evidence for the beneficial role of probiotics in children with AD. The provocative question of whether routine administration of probiotics to all infants can reverse trends in intestinal dysbiosis and dysbiosis-associated diseases remains unanswered. A large cohort study or a randomized controlled trial of probiotics in infancy with a sufficient follow-up to assess changes in dysbiosis-associated diseases is warranted and could be paradigm-shifting [100].

## 5. Conclusions

The current evidence of previous studies shows mixed results of different multi-strain and single-strain probiotics in preventing or treating AD symptoms in children, in which some probiotics were more effective than others. Our review focused on single-strain probiotic lactobacilli and has proven that certain species are promising adjuvant treatments for decreasing AD in children. It is difficult to translate the findings into a meaningful public health intervention because of the heterogeneous nature of trial outcomes and the interventions used. The results of the present systematic review and meta-analysis are not intended to replace any approved treatment for AD, such as Dupilumab, an interleukin(IL)-4 receptor alpha (IL-4Rα) antagonist [101], which the FDA approved in March 2017 for patients aged 6 years and older with moderate-to-severe AD. On the contrary, it intended to investigate the efficacy of adjuvant supplementation with single-strain probiotic lactobacilli for treating AD in children, carefully considering strain selection, treatment duration and age of treatment. More robust, well-designed clinical studies with larger samples, exact dosage, treatment time and careful strain selection to examine the effect of individual single-strain probiotic lactobacilli and multi-strain probiotics for AD as well as studies focusing on the influence of probiotics on the changes of the skin microbiota of patients with AD are warranted.

## Figures and Tables

**Table 1:** The quality assessment checklist of the 17 included clinical trials using the Joanna Briggs Institute critical appraisal tool for randomized controlled trials.

First Author, Year	Q1	Q2	Q3	Q4	Q5	Q6	Q7	Q8	Q9	Q10	Q11	Q12	Q13	Quality Index
Isolauri 2000 [41]	unclear	unclear	YES	YES	YES	YES	YES	YES	unclear	YES	YES	YES	YES	Medium-high
Kirjavainen 2003 [42]	unclear	unclear	YES	YES	NO	YES	YES	YES	unclear	YES	YES	YES	YES	Medium-high
Viljanen 2005 [43]	YES	YES	YES	YES	YES	YES	YES	YES	unclear	YES	YES	YES	YES	Excellent
Weston et al., 2005 [44]	YES	YES	YES	YES	YES	YES	YES	YES	unclear	YES	YES	YES	YES	Excellent
Brouwer 2006 [45]	unclear	unclear	YES	YES	YES	YES	YES	YES	unclear	YES	YES	YES	YES	Medium-high
Fölster-Holst 2006 [46]	unclear	unclear	YES	YES	YES	YES	YES	YES	YES	YES	YES	YES	YES	High
Grüber 2007 [47]	YES	YES	YES	YES	YES	YES	YES	YES	YES	YES	YES	YES	YES	Excellent
Woo 2010 [48]	unclear	unclear	YES	YES	YES	YES	YES	YES	unclear	YES	YES	YES	YES	Medium-high
Klewicka 2011 [49]	unclear	YES	YES	YES	YES	YES	YES	YES	unclear	YES	YES	YES	YES	High
Gore 2012 [50]	YES	YES	YES	YES	YES	YES	YES	YES	YES	YES	YES	YES	YES	Excellent
Han 2012 [51]	YES	YES	YES	YES	YES	YES	YES	YES	YES	YES	YES	YES	YES	Excellent
Wang 2015 [52]	YES	YES	YES	YES	YES	YES	YES	YES	YES	YES	YES	YES	YES	Excellent
Prakoeswa 2017 [53]	unclear	unclear	YES	YES	YES	YES	YES	YES	unclear	YES	YES	YES	YES	Medium-high
Wu 2017 [54]	unclear	unclear	YES	YES	YES	YES	YES	YES	YES	YES	YES	YES	YES	High
Ahn 2020 [55]	unclear	YES	YES	YES	YES	YES	YES	YES	unclear	YES	YES	YES	YES	High
Rather 2021 [56]	YES	YES	YES	YES	YES	YES	YES	YES	unclear	YES	YES	YES	YES	Excellent
Carucci 2022 [57]	YES	YES	YES	YES	YES	YES	YES	YES	YES	YES	YES	YES	YES	Excellent

**Table 2:** Characteristics of 17 studies using single-strain probiotic lactobacilli with the outcome measure of SCORAD index.

Reference (First Author, Year)	Population That Completed Trial	Intervention of Single-Strain Probiotic Lactobacilli		Main Findings
		Probiotic/Dosage	Duration	
Isolauri 2000 [41]	In this study, 27 patients, mean age 4.6 months, with atopic disease symptoms, divided into three equal groups. A total of 9 in two probiotic groups (Group 2: not lactobacilli) and 9 in the placebo group.	*Lacticaseibacillus* ^1^ *rhamnosus* GG (LGG), 3 × 10^8^ cfu/g	2 months	A statistically significant decrease in the SCORAD index was found in both probiotic groups compared to the placebo. Both probiotics also counteracted inflammatory responses compared to the placebo (CD4 levels were statistically significantly lower in the probiotic group compared to the placebo).
Kirjavainen 2003 [42]	In this study, 35 patients, mean age 5.5 months, with atopic disease symptoms, divided into three groups. A total of 14 in group one, 13 in group two (heat-killed LGG) and 8 in the placebo group.	*Lacticaseibacillus rhamnosus* GG, 1 × 10^9^ cfu, qd	7.5 weeks	The decrease in the SCORAD index within the viable LGG group tended to be greater than within the placebo group. The treatment with heat-inactivated LGG was associated with adverse gastrointestinal symptoms and diarrhea.
Viljanen 2005 [43]	In this study, 230 patients aged 1–12 months with atopic eczema–dermatitis syndrome, divided into three groups. A total of 80 in probiotic group 1, 76 in group 2 (multi-strain) and 74 in the placebo group.	*Lacticaseibacillus rhamnosus* GG, 5 × 10^9^ cfu, qd	4 weeks	No statistically significant effects of probiotic supplementation on mean SCORAD index reduction between groups were found. A statistically significant lower SCORAD index was observed in Ig-E sensitized infants after supplementation in the probiotic group compared to placebo.
Weston 2005 [44]	In this study, 56 patients aged 6–18 months with moderate to severe AD. A total of 26 in the probiotic group and 27 in the placebo group.	*Limosilactobacillus* ^1^ *fermentum* VRI-033 PCC, 1 × 10^9^ cfu, bid	8 weeks	A statistically significant lower SCORAD index was observed after supplementation in the probiotic group compared to the placebo. The reduction in the SCORAD index over time was significant in the probiotic group but not in the placebo group.
Brouwer 2006 [45]	In this study, 50 patients aged 1–5 months with AD, divided into three groups. A total of 33 in both single-strain probiotic lactobacilli groups (16 in group 1 and 17 in group 2) and 17 in the placebo group.	*Lacticaseibacillus rhamnosus* GG, 5 × 10^9^ cfu/g	3 months	There were no statistically significant effects of probiotic supplementation on SCORAD, sensitization, inflammatory parameters or cytokine production between groups.
		*Lacticaseibacillus rhamnosus*, 5 × 10^9^ cfu/g		
Fölster-Holst 2006 [46]	In this study, 42 patients aged 1–55 months with moderate to severe AD. A total of 21 in both the probiotic and placebo group.	*Lacticaseibacillus rhamnosus* GG, 1 × 10^10^ cfu, qd	8 weeks	No significant differences were observed between the groups with respect to the SCORAD index. No significant differences were observed between the groups with respect to other clinical symptoms (pruritus, sleep loss), the use of topical corticosteroids and antihistamines or immunological parameters.
Grüber 2007 [47]	In this study, 102 patients aged 3–12 months with mild to moderate AD. A total of 54 in the probiotic group and 48 in the placebo group.	*Lacticaseibacillus rhamnosus* GG, 5 × 10^9^ cfu, bid	12 weeks	No significant differences were observed for the SCORAD index, use of rescue medicine or increase in mean total logarithmic serum immunoglobin E after supplementation in the probiotic group compared to placebo. When stratified for age, eczema severity or use of rescue medication, no statistically significant group differences in improvement were found.
Woo 2010 [48]	In this study, 75 patients aged 2–10 years with eczema–dermatitis syndrome. A total of 41 in the probiotic group and 43 in the placebo group.	*Latilactobacillus* ^1^ *sakei* KCTC 10755BP, 5 × 10^9^ cfu, bid	12 weeks	Statistically significant lower SCORAD index, mean disease activity, proportions of patients achieving improvement and serum chemokine levels were observed after supplementation in the probiotic group compared to placebo.
Klewicka 2011 [49]	In this study, 40 patients aged 6–18 months with medium to severe AD. A total of 18 in the probiotic group and 22 in the placebo group.	*Lacticaseibacillus casei* DN-114001, 1 × 10^9^ cfu, qd	3 months	A decrease in the SCORAD index was observed in the probiotic group. Supplementation with probiotics positively affected their gut microbiota.
Gore 2012 [50]	In this study, 133 patients aged 3–6 months with AD. A total of 43 in probiotic group I, 44 in probiotic group II (not lactobacilli) and 46 in the placebo group.	*Lacticaseibacillus* ^1^ *paracasei* CNCM I-2116, 1 × 10^10^ cfu, qd	12 weeks	No significant differences were observed for the SCORAD index after supplementation in the probiotic group compared to the placebo. Results were similar when the analysis was controlled for allergen-sensitization or when only sensitized infants were analyzed.
Han 2012 [51]	In this study, 83 patients aged 1–13 years with AD. A total of 44 in the probiotic group and 39 in the placebo group.	*Lactiplantibacillus* ^1^ *plantarum* CJLP133, 5 × 10^9^ cfu, bid	12 weeks	Statistically significant mean changes in SCORAD index and lower SCORAD index, eosinophil counts, and logarithmic interferon-gamma and interleukin-4 were observed after supplementation in the probiotic group compared to placebo.
Wang 2015 [52]	In this study, 212 patients aged 1–18 years with AD and positive skin prick test, divided into 4 groups. A total of 159 in 3 probiotic groups with 55 in group 1 (single-strain), 53 in group 2 (single-strain), 51 in group 3 (both strains) and 53 in the placebo group.	*Lacticaseibacillus paracasei* GMNL-133 (Lp), 2 × 10^9^ cfu, qd	3 months	A statistically significant lower SCORAD index and interleukin-4 levels were observed after supplementation in all three probiotic groups compared to the placebo.
		*Limosilactobacillus fermentum* GM090 (Lf), 2 × 10^9^ cfu, qd		
Prakoeswa 2017 [53]	In this study, 22 patients aged 0–14 years with mild and moderate AD. A total of 12 in the probiotic group and 10 in the placebo group.	*Lactiplantibacillus plantarum* IS-10506, 1 × 10^10^ cfu, qd	12 weeks	A statistically significant lower SCORAD index and levels of interleukin-4, interferon-gamma and interleukin-17 levels were observed after supplementation in the probiotic group compared to the placebo. The ratio of forkhead box P3 to interleukin-10 was significantly higher after supplementation in the probiotic group than in the placebo group.
Wu 2017 [54]	In this study, 62 patients aged 4–48 months with AD. A total of 30 in the probiotic group and 32 in the placebo group.	*Lacticaseibacillus rhamnosus* MP108, 1 capsule ^2^, qd	8 weeks	A statistically significant lower SCORAD index was observed after supplementation in the probiotic group compared to the placebo. Mean changes from baseline declined in the probiotic and placebo groups, but no statistically significant differences were noted.
Ahn 2020 [55]	In this study, 82 patients aged 2–13 years with mild to moderate AD. A total of 41 in each group.	*Lactiplantibacillus pentosus* ^3^, 1 × 10^10^ cfu, bid	12 weeks	No significant difference was found in the reduction in the SCORAD index after supplementation in the probiotic group compared to the placebo. However, a statistically significant reduction in the SCORAD index was observed in the subgroup of Immunoglobulin E-sensitized AD compared to placebo.
Rather 2021 [56]	In this study, 58 patients aged 3–18 years with mild to moderate AD divided into three groups. A total of 16 in the probiotic group, 22 in group II (received dead cells) and 20 in the placebo group.	*Latilactobacillus sakei* proBio65, 10^10^ cfu, qd	12 weeks	A statistically significant lower SCORAD total index was observed after supplementation in the probiotic group and the group receiving non-viable cells compared to the placebo. Statistically significant increase in skin sebum in the probiotic group as well as in the group that received non-viable cells.
Carucci 2022 [57]	In this study, 91 patients aged 6–36 months with AD. A total of 46 in probiotic group A and 45 in the placebo group.	*Lacticaseibacillus rhamnosus* GG, 10^10^ cfu, qd	12 weeks	A statistically significant higher rate of participants in the probiotic group after supplementation achieved the minimum clinically important difference in the SCORAD index compared to the placebo. The probiotic group also observed a beneficial modulation of the gut and skin microbiome.

Bid: Twice per day; qd: once per day; ^1^ new nomenclature [12]; ^2^ cfu not reported; ^3^ strain not reported.

## Data Availability

Data are available from the corresponding author on request.

## Supplementary Materials

The following supporting information can be downloaded at: https://www.mdpi.com/article/10.3390/pharmaceutics15041256/s1. Supplementary S1: PRISMA 2020 Checklist.
